# Safety, tolerability, and preliminary efficacy of seltorexant versus quetiapine extended release as adjunctive therapy in major depressive disorder: a randomized, flexible-dose, 6-month, parallel-group, exploratory study

**DOI:** 10.1093/ijnp/pyag009

**Published:** 2026-03-09

**Authors:** Christine Pinter, Michael E Thase, Roger S McIntyre, Kimberly Cooper, Haiyan Xu, Gahan Pandina, Adam Savitz, Wayne C Drevets

**Affiliations:** Neuroscience Clinical Development, Johnson & Johnson, Titusville, NJ, United States; Department of Psychiatry, Perelman School of Medicine, University of Pennsylvania, Philadelphia, PA, United States; Mood & Anxiety Disorders Section, Corporal Michael J. Crescenz Veterans Affairs Medical Center, Philadelphia, PA, United States; Department of Psychiatry, University of Toronto, Toronto, ON, Canada; Department of Pharmacology and Toxicology, University of Toronto, Toronto, ON, Canada; Department of Statistics & Decision Sciences, Johnson & Johnson, Spring House, PA, United States; Department of Statistics & Decision Sciences, Johnson & Johnson, Titusville, NJ, United States; Neuroscience Clinical Development, Johnson & Johnson, Titusville, NJ, United States; Neuroscience Clinical Development, Johnson & Johnson, Titusville, NJ, United States; Neuroscience Clinical Development, Johnson & Johnson, San Diego, CA, United States

**Keywords:** antidepressant, major depressive disorder, orexin-2 receptor, seltorexant, adjunctive treatment

## Abstract

**Importance:**

Seltorexant, a selective orexin-2 receptor (OX2R) antagonist, has demonstrated antidepressant effects in major depressive disorder (MDD), particularly among patients with higher baseline insomnia symptoms.

**Objective:**

To investigate flexibly dosed seltorexant vs flexibly dosed quetiapine extended release (quetiapine-XR) as adjunctive treatment to a selective serotonin (SSRI) or serotonin-norepinephrine (SNRI) reuptake inhibitor.

**Setting:**

Outpatient.

**Design:**

Randomized, active-controlled, multicenter, exploratory phase 2 study with screening (≤4 weeks), double-blind treatment (24 weeks), and post-treatment follow-up (2 weeks) phases.

**Participants:**

Patients with MDD and inadequate response to 1-3 SSRIs/SNRIs, including an ongoing SSRI/SNRI, in the current depressive episode.

**Interventions:**

Flexibly dosed seltorexant (20 or 40 mg) or quetiapine-XR (150 or 300 mg, with 2-day initial dosing of 50 mg) once daily as adjunctive therapy to an SSRI/SNRI. Randomization (1:1) was stratified by baseline Insomnia Severity Index total score (≥15 vs <15). Safety, tolerability, and preliminary efficacy were evaluated.

**Main outcomes and measures:**

Primary efficacy endpoint was time to all-cause study drug discontinuation. Secondary efficacy endpoints included change in Montgomery-Åsberg Depression Rating Scale (MADRS) total score. Subgroup analyses included MADRS change by mode dose (MD; most frequent daily dose received by a patient during the study). Safety and tolerability also were assessed.

**Results:**

Time to all-cause discontinuation (estimated 25^th^ percentile [80% CI]: seltorexant, 62 [38, 83] days vs quetiapine-XR, 42 [35, 61] days; hazard ratio [80% CI]: 0.83 [0.6, 1.2]) and all-cause discontinuation (seltorexant, 41.2% vs quetiapine-XR, 47.1%; 2-sided *P* = .5355) did not differ significantly between treatment groups. For the seltorexant 20-mg MD group, MADRS total scores consistently improved over time and reductions were numerically greater at weeks 18 and 24 versus the seltorexant 40-mg MD and the combined quetiapine-XR groups, and patients with higher baseline insomnia symptoms had greater improvement in MADRS total score, consistent with prior studies showing efficacy at 20 but not 40 mg. Treatment-emergent adverse event rates were 65.4% for seltorexant and 80.8% for quetiapine-XR.

**Conclusions and relevance:**

Results support the favorable tolerability and preliminary efficacy of seltorexant 20 mg daily as adjunctive treatment in patients with MDD, especially those with insomnia symptoms, and suggest potential approaches to differentiate seltorexant from quetiapine-XR in future adequately powered studies.

**Trial registration:**

Clinicaltrials.gov  **identifier:** NCT03321526.

Significance statement Seltorexant has demonstrated antidepressant effects in major depressive disorder (MDD). In this exploratory study, patients with MDD and inadequate response to current antidepressant therapy received flexibly dosed seltorexant (20 or 40 mg) or quetiapine extended release (quetiapine-XR; 150 or 300 mg) as adjunctive treatment. All-cause discontinuations were comparable between seltorexant and quetiapine-XR, but discontinuations due to potential treatment-related reasons were fewer for seltorexant. Seltorexant 20-mg mode dose (most frequent daily dose received by a patient during the study) showed consistently improved Montgomery–Åsberg Depression Rating Scale (MADRS) total scores over time and numerically greater score reductions at weeks 18 and 24 versus seltorexant 40-mg mode dose and combined quetiapine-XR, and those with higher baseline insomnia symptoms had even greater improvements in MADRS total score. No new safety signals were identified. Findings support the safety and preliminary efficacy of seltorexant as a novel adjunctive treatment for MDD, especially among those with insomnia symptoms.

## Introduction

Inadequate (partial) response to pharmacologic treatment for major depressive disorder (MDD) is common.^1^ In the Sequenced Treatment Alternatives to Relieve Depression (STAR^*^D) study, only 28% of patients achieved remission (HAM-D ≤ 7) during first-line treatment with a selective serotonin reuptake inhibitor (SSRI).^2^ For patients with inadequate response to an optimized trial of first-line antidepressant treatment (ADT), switching the ADT (within or across drug classes), adding a second ADT, or therapy with a non-ADT is recommended.^3^ Adjunctive treatment with second-generation antipsychotics (eg, quetiapine [Seroquel XR])^4^^,^^5^—the only adjunctive treatments approved in both the United States and European Union—is associated with significant improvements in treatment response and remission; however, side effects (eg, weight gain) may limit use in clinical practice.^6–9^ Thus, a clinical need persists for adjunctive strategies that offer greater efficacy and safety for patients with MDD.

The orexinergic system plays an important role in maintaining sleep–wake states, energy regulation, stress response, and consummatory or reward-directed behavior, and dysregulation of orexin function is associated with neuropsychiatric diseases such as panic and anxiety disorders, and depression.^10–13^ In completed seltorexant clinical trials, selective orexin-2 receptor (OX2R) antagonism improved depression symptoms in patients with MDD, particularly those with heightened arousal as manifested by insomnia symptoms.^14–18^ For example, seltorexant was efficacious as adjunctive treatment (with and without insomnia symptoms) in 3 studies (NCT02476058,^14^ NCT03227224,^15^^,^^19^ and NCT04533529^17^) and as monotherapy in another study (NCT03374475).^16^ Notably, a larger treatment difference was observed between 20-mg seltorexant and placebo in patients with significant insomnia symptoms than in patients with mild or no insomnia symptoms.^15^^,^^16^

The primary objective of this study was to determine the time to discontinuation, and antidepressant effects, of flexibly dosed seltorexant vs flexibly dosed quetiapine extended release (quetiapine-XR) as adjunctive treatment to an SSRI/selective norepinephrine reuptake inhibitor (SNRI) in patients with MDD. Quetiapine-XR was included as an active comparator since both seltorexant^14^^,^^15^^,^^20^^,^^21^ and quetiapine-XR^6^^,^^22^^,^^23^ induce sleep. This design allowed for evaluation of potential differentiating clinical features between seltorexant and quetiapine-XR as adjunctive agents to an SSRI/SNRI in the current depressive episode. Other objectives included determining whether improvement in depressive symptoms with seltorexant could be maintained longer-term and evaluating the long-term safety of seltorexant. This study was devised as a pilot to guide the design of a larger trial (NCT04513912), aimed at comparing the effectiveness of adjunctive therapy with seltorexant versus quetiapine-XR in adult and elderly patients with MDD with insomnia symptoms who responded inadequately to antidepressant therapy.

## Methods

### Study design

**Figure 1:**
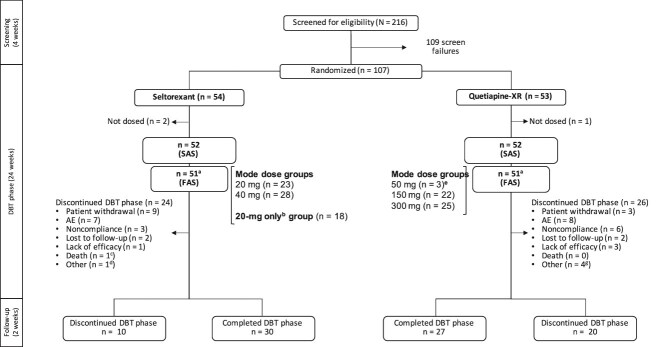
Patient flow diagram. Abbreviations: 20-mg only, no change in seltorexant 20-mg dose during the study; AE, adverse event; DBT, double-blind treatment; FAS, full analysis set (received ≥1 dose of study drug); mode dose, most frequent daily dose received by a patient during the study; SAS, safety analysis set; XR, extended release. ^a^One patient in each treatment group received a potentially defective investigational product, was withdrawn from the DBT phase (listed as “other”), and was excluded from the FAS. ^b^Seltorexant 20-mg–only group comprised 18 patients who remained on seltorexant 20 mg without any adjustments throughout the study. ^c^The death was considered not treatment emergent and unrelated to the study drug. The patient had a history of daily alcohol use but did not have alcohol use disorder reported at screening. The patient developed a subarachnoid hemorrhage and subdural hematoma after falling while intoxicated (alcohol level: 308 mg/dL) on study day 51, 3 days after the last recorded dose of seltorexant, and died on study day 53. ^d^Received potentially defective investigational product (*n* = 1). ^e^The 3 patients in the 50-mg quetiapine-XR mode-dose group discontinued treatment early due to AE, and prior to their treatment discontinuation: 1 patient only received 2 doses of 50-mg quetiapine-XR; 1 patient received 2 doses of 50-mg quetiapine-XR and 1 dose of 150-mg quetiapine-XR; and 1 patient received 2 doses of 50-mg quetiapine-XR and 2 doses of 150-mg quetiapine-XR. Data for the patients in the 50-mg quetiapine-XR mode-dose group were added to the 150-mg quetiapine-XR mode-dose group for graphical display of the by-mode-dose analysis of Montgomery–Åsberg depression rating scale data. Mode dose was not calculated for the patient who discontinued after receiving 1 50-mg dose of quetiapine-XR and 1150 mg dose of quetiapine-XR. ^f^Received potentially defective investigational product (*n* = 1), non-compliance with antidepressant therapy (*n* = 2), and randomized in error (*n* = 1). Although doses could be adjusted down based on efficacy and tolerability after being increased to a higher dose, only 5 patients in each treatment group had their doses lowered; 2 of these patients in each group returned to the higher dose.

This exploratory, double-blind, randomized, active-controlled, flexible-dose, phase 2 study (NCT03321526) was conducted at 38 centers in the United States between December 13, 2017, and June 27, 2019, and included 3 study phases: screening (≤4 weeks), double-blind treatment (DBT; 24 weeks), and post-treatment follow-up (2 weeks) (Figure 1). This study was conducted in accordance with the ethical principles of the Declaration of Helsinki and adhered to the International Conference on Harmonization’s Good Clinical Practice guidelines and applicable regulatory standards. The study protocol and amendments were reviewed and approved by Independent Ethics Committee/Institutional Review Board(s). Patients provided written informed consent before enrollment and study participation.

### Study drugs

Eligible patients were randomized 1:1 to receive flexibly dosed seltorexant (20 or 40 mg) or quetiapine-XR (150 or 300 mg, with 2-day initial dosing of 50 mg) once daily as adjunctive therapy to an SSRI/SNRI to which they had an inadequate response in the current depressive episode, including at the time of screening (Supplementary Figure 1). Patients continued taking this SSRI/SNRI throughout the study at the same dose and time as before study enrollment.

Randomized treatment assignment was performed centrally using an Interactive Web Response System, balanced using randomly permuted blocks and stratified by the presence or absence of significant insomnia symptoms at baseline (based on Insomnia Severity Index [ISI] clinician version total score ≥ 15 vs <15, respectively). Patients self-administered the assigned, blinded study drug once daily at bedtime, approximately 3 hours after their last meal. After DBT phase completion or early withdrawal, patients completed a follow-up visit within 7-14 days after the last study-drug dose.

### Patients

Outpatients aged 18-70 years with MDD and an inadequate response to 1-3 antidepressants, including an ongoing SSRI/SNRI, in the current depressive episode were enrolled. Prohibited medications included monoamine oxidase inhibitors and antipsychotics (except study-assigned quetiapine-XR) within 4 weeks before day 1 until the follow-up visit, and benzodiazepines, hypnotics (eg, zolpidem, zopiclone, zaleplon, eszopiclone, suvorexant, and ramelteon), sedating antidepressants (eg, doxepin, trazodone, mirtazapine, and tricyclic antidepressants), sedating antihistamines including over-the-counter hypnotics (eg, diphenhydramine, doxylamine, and hydroxyzine), and melatonin ≥7 days before day 1 until the follow-up visit. Additional inclusion and exclusion criteria, as well as prohibited medications and food supplements, are described in Supplemental Materials.

### Efficacy and safety evaluations

Primary endpoint was time to all-cause study drug discontinuation (number of days from first to last study-drug dose), estimated by the Kaplan–Meier method and summarized (number of discontinuations, number of censored events, median, and 25th and 75th percentiles, if estimable) by treatment group. Time to all-cause discontinuation was selected as the primary outcome because of tolerability concerns during long-term quetiapine-XR use. In a placebo-controlled study of quetiapine-XR monotherapy as maintenance therapy in patients with MDD, the discontinuation rate was >50%.^24^ Secondary efficacy endpoints, including sustained response and sustained remission, and corresponding descriptions, are summarized in Supplementary Table 1.

Safety and tolerability were evaluated by assessing treatment-emergent adverse events (TEAEs), including TEAEs of special interest (cataplexy, sleep paralysis, and complex sleep-related behaviors), clinical laboratory tests with insulin sensitivity and metabolic parameters, electrocardiogram data, vital sign measurements, and physical examinations; suicide risk using the Columbia Suicide Severity Rating Scale (C-SSRS)^25^; and sexual function using the Arizona Sexual Experience Scale (ASEX).^26^

### Statistical methods

#### Sample size determination and analysis sets

Patients were classified into all-randomized, full (FAS), and safety (SAS) analysis sets. Corresponding details and sample size determination are in Supplemental Materials.

#### Efficacy and safety analyses

Efficacy analyses were based on the FAS. Primary endpoint (time to all-cause study drug discontinuation)—presented as number (%) of events and 25th percentile (80% confidence interval [CI])—was assessed using a stratified log-rank test to evaluate treatment differences (non-stratified log-rank also performed as a sensitivity analysis). Unless otherwise stated, all efficacy analyses were preplanned. Hazard ratio of all-cause discontinuation and its 80% CI were estimated based on the Cox proportional hazards model, with treatment and baseline insomnia status as factors.

A mixed-model-for-repeated-measures (MMRM) analysis of the Montgomery–Åsberg Depression Rating Scale (MADRS) total score^27^ (Supplementary Table 1) change from baseline was used. The MMRM included time, treatment, baseline insomnia status, and treatment-by-time interaction as factors, and baseline MADRS total score as a covariate; follow-up phase data were not used. An 80% CI for the difference in least-squares (LS) means was calculated. A similar MMRM model was applied to changes in MADRS-6 subscale^28^ (apparent sadness, reported sadness, inner tension, lassitude, inability to feel, and pessimistic thoughts), Hamilton Anxiety Rating Scale (HAM-A),^29^ Quality of Life in Depression Scale (QLDS),^30^ Patient-Reported Outcomes Measurement Information System-Sleep Disturbance Short Form 8a (PROMIS-SD),^31^ PROMIS-Sleep Related Impairment Short Form 8a (PROMIS-SRI),^31^ Symptoms of Major Depressive Disorder Scale (SMDDS),^32^^,^^33^ Symbol Digit Modalities Test (SDMT),^34^ and Trail Making Test-Part B (TMT-Part B)^35^ (Table 1), with their corresponding baseline scores as covariates. In addition, response (≥50% improvement in MADRS total score from baseline) over time and remission (MADRS ≤12) over time (at week 2, 4, 6 12, 18, or 24) were assessed. Patients with missing values were imputed as non-responders.

**Table 1:** Demographics and baseline characteristics (full analysis set).

	Seltorexant *n* = 51	Quetiapine-XR *n* = 51	Total *n* = 102
Age, years	55.3 (9.77)	53.4 (10.84)	54.4 (10.31)
Female sex, *n* (%)	34 (66.7)	34 (66.7)	68 (66.7)
Race, *n* (%)			
White	35 (68.6)	36 (70.6)	71 (69.6)
Black or African American	14 (27.5)	14 (27.5)	28 (27.5)
Native Hawaiian or other Pacific Islander	2 (3.9)	0 (0.0)	2 (2.0)
Multiple	0 (0.0)	1 (2.0)	1 (1.0)
Age when diagnosed with MDD, years	38.3 (15.53)	37.5 (14.41)	37.9 (14.91)
Duration of current depressive episode, weeks	22.8 (14.66)	22.5 (14.58)	22.6 (14.55)
Baseline MADRS total score	32.9 (5.34)	33.9 (4.44)	33.4 (4.91)
Baseline CGI-S score	4.6 (0.72)	4.8 (0.76)	4.7 (0.74)
Baseline ISI total score per electronic data capture, *n* (%)			
≥15	33 (64.7)	34 (66.7)	67 (65.7)
<15	18 (35.3)	17 (33.3)	35 (34.3)
Current antidepressant type, *n* (%)			
SSRI	39 (76.5)	35 (68.6)	74 (72.5)
SNRI	12 (23.5)	16 (31.4)	28 (27.5)
Antidepressant treatment history^a^, *n* (%)			
1	48 (94.1)	45 (88.2)	93 (91.2)
≥2	3 (5.9)	6 (11.8)	9 (8.8)
Number of major depressive episodes in lifetime, including current episode, *n* (%)			
1	0 (0.0)	1 (2.0)	1 (1.0)
2	9 (17.6)	2 (3.9)	11 (10.8)
≥3	42 (82.4)	48 (94.1)	90 (88.2)

Abbreviations: CGI-S, Clinical Global Impression–Severity; ISI, Insomnia Severity Index; MADRS, Montgomery–Asberg Depression Rating Scale; MDD, major depressive disorder; MGH-ATRQ, Massachusetts General Hospital–Antidepressant Treatment Response Questionnaire; SNRI, serotonin–norepinephrine reuptake inhibitor; SSRI, selective serotonin reuptake inhibitor; XR, extended release.

Data shown are mean (SD) unless otherwise indicated.

a Number of medications with inadequate response taken for ≥4 weeks during the current episode based on MGH-ATRQ.

As sensitivity analyses, changes in MADRS, MADRS-6, and HAM-A scores were analyzed using an analysis of covariance (ANCOVA) model with last-observation-carried-forward data. Analysis of covariance on ranks was used to analyze change in Clinical Global Impression-Severity (CGI-S)^36^ and Patient Global Impression-Severity (PGI-S).^37^ Kaplan–Meier analysis of time to potentially treatment-related discontinuation was performed as a secondary analysis (planned pre-database lock), where patients who discontinued because of potentially non–treatment-related reasons (eg, moved, changed job, unable to pay for SSRI/SNRI) were censored at the last study-drug dose date. Time to all-cause study drug discontinuation; changes in MADRS, MADRS-6, and PROMIS-SD scores; and response and remission over time were summarized by mode dose (MD; daily dose received most frequently by a patient during the study): seltorexant, 20-mg and 40-mg MD groups (MDGs); quetiapine-XR, 50-mg, 150-mg, and 300-mg MDGs. Further evaluations were conducted by separately analyzing seltorexant 20 mg (20-mg only [no change in dose during study] or 20-mg MD) and 40-mg MD. Except analysis of MADRS total score change by MD, other analyses by MD, by final dose, or for the seltorexant 20-mg–only group were post hoc. A causal inference analysis, adjusting for potential bias in estimates of MD treatment effects (MADRS total score change from baseline over time) using propensity score weighting also was performed post hoc (Supplemental Materials).

Safety assessments collected during the study were summarized. For metabolic laboratory parameters, 95% CI for within-treatment–group change from baseline were calculated post hoc. Safety analyses by MD groups, arithmetic mean summaries of HOMA-IR and HOMA-%B, and ASEX summaries by sex were post hoc.

## Results

### Patient disposition and baseline characteristics

Of 216 patients screened, 107 were randomly assigned to receive seltorexant (*n* = 54) or quetiapine-XR (*n* = 53) during DBT (Figure 1). The SAS included 104 patients (52/treatment group). The FAS included 102 patients (51/treatment group) since 2 patients (1/treatment group) were excluded because they received a potentially defective investigational product and were discontinued early. Most commonly, discontinuations were due to AEs (15 [14.0%]), patient withdrawal of consent (12 [11.2%]), and study drug non-compliance (9 [8.4%]).

Demographics and baseline characteristics were comparable between treatment groups (Table 1). In the FAS, most patients were female (66.7%) and White (69.6%); 27.5% were Black or African American. Mean (SD) patient age was 54.4 (10.31) years. Mean (SD) current depressive episode duration was 22.6 (14.55) weeks, and 88.2% of patients experienced ≥3 lifetime major depressive episodes. Mean (SD) baseline MADRS total score was 33.4 (4.91), consistent with moderate-to-severe depression, and 65.7% of patients had a baseline ISI total score ≥ 15. Most (91.2%) patients had an inadequate response to only 1 antidepressant in the current episode, and 72.5% were taking an SSRI.

### Primary endpoint

**Figure 2:**
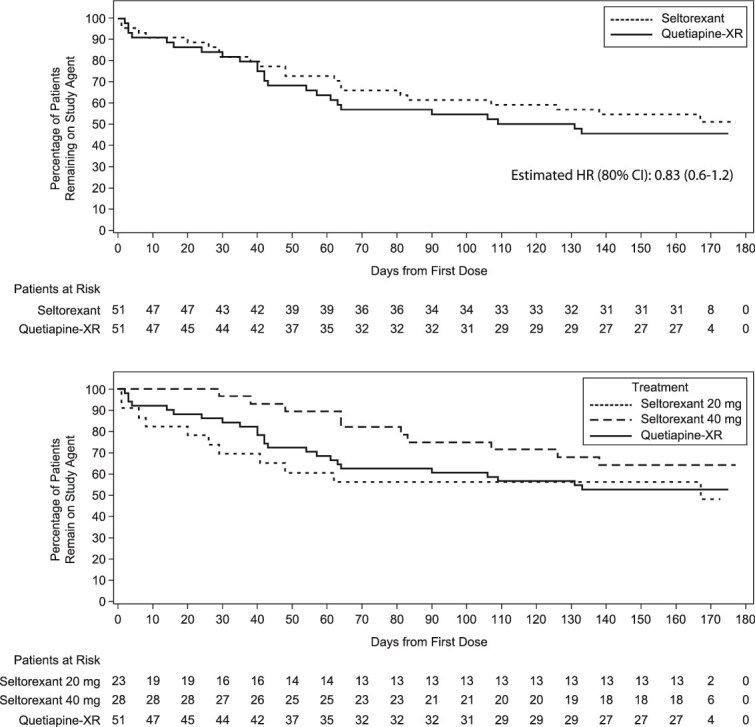
Time to all-cause discontinuation of study drug by treatment group (full analysis set) and by mode-dose group. Abbreviations: CI, confidence interval; HR, hazard ratio; XR, extended release.

Time to all-cause discontinuation numerically favored seltorexant but did not significantly differ between treatment groups (estimated 25^th^ percentile [80% CI]: 62 [38, 83] days for seltorexant vs 42 [35, 61] days for quetiapine-XR; hazard ratio [80% CI]: 0.83 [0.6, 1.2]; Figure 2 and Supplementary Figure 2 [stratification by baseline ISI total score]). All-cause discontinuation rates also did not differ significantly between groups (seltorexant, 41.2% [21/51]; quetiapine-XR, 47.1% [24/51]) when stratified by baseline ISI total score (2-sided; *P* = .5355) or not (2-sided; *P* = .5160). All-cause discontinuation examined by final dose, MD, and 20-mg–only dose showed similar trends for all analyses. In a post hoc analysis, the seltorexant 20-mg MDG had a comparable time to all-cause discontinuation as the combined quetiapine-XR group—partly because all early discontinuations were among patients receiving low-dose treatment (seltorexant 20 mg; quetiapine-XR 50 or 150 mg), and the dose could not be raised until Day 14 (Figure 2).

### Pre-planned sensitivity analysis for primary endpoint

**Figure 3:**
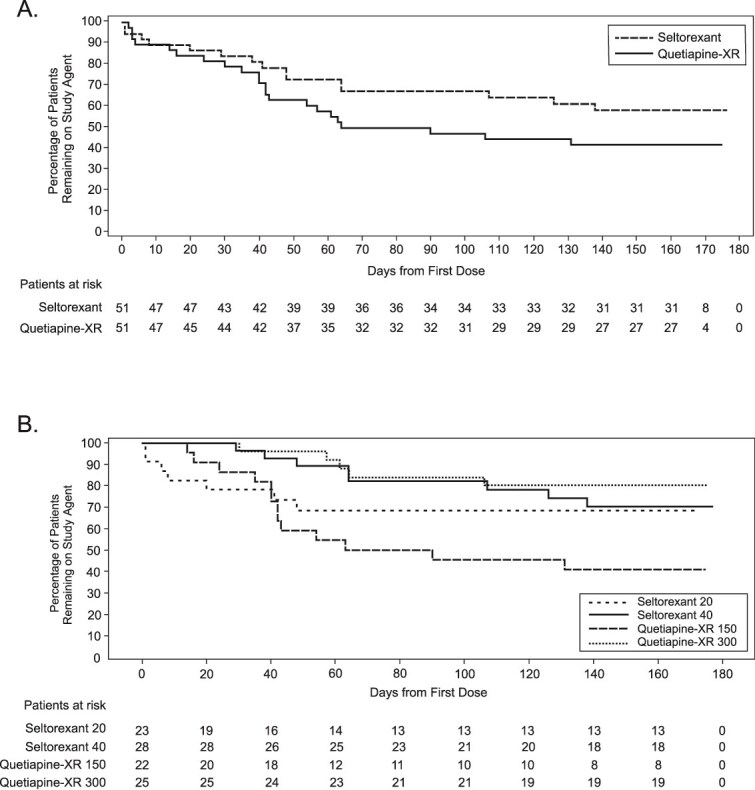
Time to potentially treatment-related discontinuation by treatment group (A) and mode-dose group (B) (full analysis set). Abbreviation: XR, extended release.

Eight patients (seltorexant, *n* = 6; quetiapine-XR, *n* = 2) discontinued for potentially non–treatment-related reasons, and 37 patients (seltorexant, *n* = 15; quetiapine-XR, *n* = 22) discontinued for potentially treatment-related reasons (between-group difference: stratified [2-sided; *P* = .1973] and not stratified [2-sided; *P* = .1885] by baseline ISI status; Figure 3). Time to potentially treatment-related discontinuation was longer with seltorexant than quetiapine-XR (estimated 25^th^ percentile [80% CI]: 107 [48 to –] days for seltorexant vs 42 [35 to 61] days for quetiapine-XR).

### Secondary endpoints

#### MADRS total score

**Figure 4:**
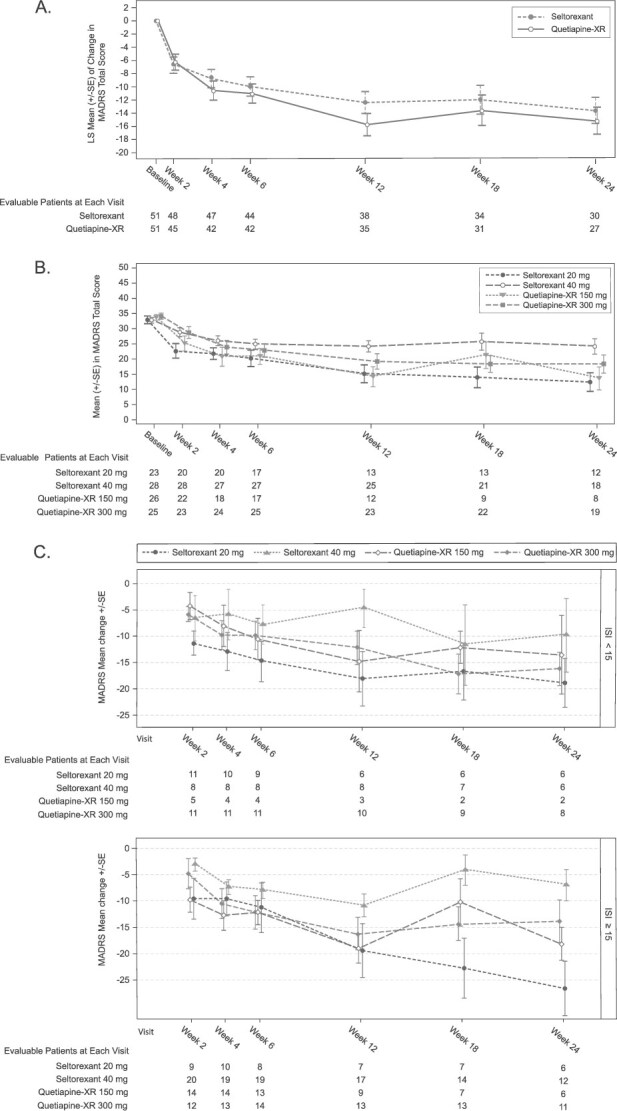
Change in MADRS total score over time by treatment group (A), mode-dose group (B), and baseline ISI total score and mode-dose group (C) (full analysis set). Abbreviations: ISI, insomnia severity index; MADRS, Montgomery–Åsberg depression rating scale; SE, standard error; XR, extended release. Data for the patients in the 50-mg quetiapine-XR mode-dose group were added to the 150-mg quetiapine mode-dose group for graphical display of the by-mode-dose analysis of MADRS data for graphical display. Mode dose was not calculated for the patient who discontinued after receiving 1 50-mg dose of quetiapine-XR and 1150-mg dose of quetiapine-XR.

Mean (SD) MADRS total score change from baseline to week 24 was comparable between seltorexant (−13.8 [14.13]) and quetiapine-XR (−15.4 [10.57]; LS mean difference [80% CI]: 1.6 [−2.0, 5.2]) (Table 2**,**  Figure 4A). However, the seltorexant 20-mg MDG consistently improved over time compared with the seltorexant 40-mg and combined quetiapine-XR groups (Figure 4B). At weeks 18 and 24, mean (SD) change in MADRS was similar between the seltorexant 20-mg MD (−19.9 [14.06] and −22.7 [12.09], respectively) and seltorexant 20-mg–only (−21.4 [14.66] and −23.2 [12.43], respectively) groups, and changes for both groups were numerically greater than for the combined quetiapine-XR group (−14.1 [11.14] and −15.4 [10.57], respectively). Differences between seltorexant 20- and 40-mg MDGs may have been confounded by the effect/amount of improvement at week 2 before starting dose adjustment and other potential factors. In a post hoc analysis adjusted for propensity score weighting, LS mean differences (80% CI) between the seltorexant 20-mg MDG and combined quetiapine-XR group at weeks 18 (−4.5–point difference [−9.4; 0.3]) and 24 (−3.4–point difference [−7.8; 1.0]) favored seltorexant and remained clinically meaningful (difference > 2 points). Mean MADRS total score changes from baseline were similar for the 2 quetiapine-XR MDGs in unweighted and weighted analyses. Among patients with ISI total score ≥ 15, those in the seltorexant 20-mg MDG had numerically greater improvement in MADRS total score than those in the seltorexant 40-mg MDG or quetiapine-XR group (Figure 4C).

**Table 2:** Secondary endpoint results (full analysis set).

	Seltorexant							
	*n*	20-mg MD	*n*	40-mg MD	*n*	20 mg only	Seltorexant *n* = 51	Quetiapine-XR *n* = 51
MADRS total score								
Baseline, mean (SD)	23	32.9 (5.95)	28	32.9 (4.88)	18	32.4 (6.46)	32.9 (5.34)	33.9 (4.44)
Change from baseline to week 12, mean (SD)	13	−18.8 (12.54)	23	−8.9 (9.34)	11	−20.3 (12.81)	−12.3 (11.41)^b^	−15.6 (9.80)^b^
LS mean difference^a^ at week 12 (80% CI)							3.4 (0.3, 6.4)^b^	
Change from baseline to week 24, mean (SD)	12	−22.7 (12.09)	18	−7.9 (12.42)	10	−23.2 (12.43)	−13.8 (14.13)	−15.4 (10.57)
LS mean difference^a^ at week 24 (80% CI)							1.6 (−2.0, 5.2)^c^	
MADRS-6 score								
Baseline, mean (SD)	23	22.6 (3.56)	28	21.8 (3.49)		—	22.2 (3.51)	22.8 (3.20)
Change from baseline to week 12, mean (SD)	13	−11.8 (9.34)	25	−5.3 (6.69)		—	−7.5 (8.18)^b^	−10.4 (7.68)^b^
LS mean difference^a^ at week 12 (80% CI)						—	2.5 (0.3, 4.8)^b^	
Change from baseline to week 24, mean (SD)	12	−14.9 (7.93)	18	−5.2 (8.38)		—	−9.1 (9.40)^c^	−10.3 (8.59)^c^
LS mean difference^a^ at week 24 (80% CI)						—	0.7 (−1.9, 3.2)^c^	
PROMIS-SD T-score								
Baseline, mean (SD)	23	59.5 (8.77)	28	62.9 (7.57)		—	61.3 (8.23)	61.7 (7.33)
Change from baseline to week 12, mean (SD)	13	−12.4 (10.76)	24	−6.0 (9.13)		—	−8.22 (10.1)^d^	−11.87 (10.7)^d^
LS mean difference^a^ at week 12 (80% CI)						—	4.24 (1.71, 6.77)^d^	
Change from baseline to week 24, mean (SD)	12	−17.8 (12.11)	18	−7.2 (9.59)		—	−11.45 (11.72)^c^	−12.91 (7.88)^c^
LS mean difference^a^ at week 24 (80% CI)							1.82 (−0.96, 4.59)^c^	
PROMIS-SD raw score								
Baseline, mean (SD)	23	28.4 (7.36)	28	31.5 (6.35)		—	30.1 (6.94)	30.6 (6.23)
Change from baseline to week 12, mean (SD)	13	−10.2 (8.3)	24	−5.1 (8.11)		—	−6.9 (8.43)^d^	−10.2 (8.99)^d^
LS mean difference^a^ at week 12 (80% CI)							3.8 (1.7, 5.9)^d^	
Change from baseline to week 24, mean (SD)	12	−14.3 (9.71)	18	−6.3 (7.87)		—	−9.5 (9.37)^c^	−11.2 (6.70)^c^
LS mean difference^a^ at week 24 (80% CI)							2.0 (−0.3, 4.2)^c^	

Abbreviations: CI, confidence interval; LS, least squares; MADRS, Montgomery–Asberg Depression Rating Scale; MD, mode dose, most frequently received daily dose received by a patient during the study; PROMIS-SD, Patient Reported Outcome Measurement Information System-Sleep Disturbance; SD, standard deviation; XR, extended release.

a Based on mixed model for repeated-measures analyses.

b Selorexant, *n* = 35; quetiapine-XR, *n* = 38.

c Selorexant, *n* = 30; quetiapine-XR, *n* = 27.

d Selorexant, *n* = 37; quetiapine-XR, *n* = 36.

#### MADRS-6 score

Mean (SD) MADRS-6 score change from baseline to week 24 was -9.0 (9.40) for the combined seltorexant group and −9.7 (8.59) for the combined quetiapine-XR group (LS mean difference [80% CI]: 0.7 [−1.9, 3.2]; Table 2). The seltorexant 20-mg MDG consistently improved over time compared with the seltorexant 40-mg MDG and combined quetiapine-XR group (Table 2).

#### Sustained response and sustained remission

Analysis of sustained response did not show an overall greater benefit for seltorexant versus quetiapine-XR. Sustained response was achieved by 13.2% of the combined seltorexant group and 22.2% of the combined quetiapine-XR group, and sustained remission was achieved by 13.2% of the seltorexant group vs 19.4% of the quetiapine-XR group.

#### Response

In post hoc analyses, response rates over time for the seltorexant 20-mg MDG were comparable to or better than those for the combined quetiapine-XR group at most timepoints and were numerically greater at week 24 (30.4% vs 21.6%; Table 3). At weeks 18 and 24, rates were numerically greater for the seltorexant 20-mg–only group (38.9% and 33.3%, respectively) vs the combined quetiapine-XR group (27.5% and 21.6%, respectively).

**Table 3:** Patients who achieved response^a^ over time (full analysis set).

Responders, *n* (%)^a^	Seltorexant				Quetiapine-XR *n* = 51
	20-mg MD *n* = 23	40-mg MD *n* = 28	20 mg only *n* = 18	Seltorexant *n* = 51	
Week 2	6 (26.1)	1 (3.6)	5 (27.8)	7 (13.7)	7 (13.7)
Week 4	5 (21.7)	2 (7.1)	4 (22.2)	7 (13.7)	12 (23.5)
Week 6	6 (26.1)	4 (14.3)	5 (27.8)	10 (19.6)	14 (27.5)
Week 12	6 (26.1)	5 (17.9)	6 (33.3)	11 (21.6)	17 (33.3)
Week 18	7 (30.4)	4 (14.3)	7 (38.9)	11 (21.6)	14 (27.5)
Week 24	7 (30.4)	4 (14.3)	6 (33.3)	11 (21.6)	11 (21.6)

Abbreviations: 20 mg only, no change in seltorexant 20-mg dose during the study; MADRS, Montgomery–Asberg Depression Rating Scale; MD (mode dose), most frequently received daily dose received by a patient during the study; XR, extended release.

a Response defined as ≥50% improvement in MADRS total score from baseline to the given time point. Patients with missing values at a given time point were imputed as non-responders.

#### Remission

At weeks 12, 18, and 24, remission rates were 15.7%, 19.6%, and 19.6%, respectively, for the combined seltorexant group and 23.5%, 19.6%, and 21.6%, respectively, for the combined quetiapine-XR group (Table 4). In a post hoc analysis over time, remission rates for the seltorexant 20-mg MDG and combined quetiapine-XR group were consistently numerically greater than for the seltorexant 40-mg MDG (Table 4). Rates were numerically greater for the seltorexant 20-mg–only group than for both quetiapine-XR MDGs at all timepoints, except week 4.

**Table 4:** Patients who achieved remission^a^ over time (full analysis set).

Remitters, *n* (%)^a^	Seltorexant			Seltorexant *n* = 51	Quetiapine-XR *n* = 51
	20-mg MD *n* = 23	40-mg MD *n* = 28	20 mg only *n* = 18		
Week 2	3 (13.0)	1 (3.6)	3 (16.7)	4 (7.8)	4 (7.8)
Week 4	2 (8.7)	1 (3.6)	2 (11.1)	3 (5.9)	8 (15.7)
Week 6	4 (17.4)	1 (3.6)	4 (22.2)	5 (9.8)	9 (17.6)
Week 12	6 (26.1)	2 (7.1)	6 (33.3)	8 (15.7)	12 (23.5)
Week 18	7 (30.4)	3 (10.7)	7 (38.9)	10 (19.6)	10 (19.6)
Week 24	7 (30.4)	3 (10.7)	6 (33.3)	10 (19.6)	11 (21.6)

Abbreviations: 20 mg only, no change in seltorexant 20-mg dose during the study; MADRS, Montgomery–Åsberg Depression Rating Scale; MD (mode dose), most frequently received daily dose received by a patient during the study; XR, extended release.

a Remission was defined as an MADRS total score of ≤12 at a given time point. Patients with missing values at a given time point were imputed as non-remitters.

#### Sleep disturbance and impairment

Mean (SD) PROMIS-SD T-score change from baseline to week 24 was comparable for the combined seltorexant and combined quetiapine-XR groups (−11.45 [11.72] and −12.91 [7.88], respectively; LS mean difference [80% CI]: 1.82 [−0.96, 4.59]) (Table 2). Likewise, mean (SD) PROMIS-SRI T-score change from baseline to week 24 was similar for the seltorexant and quetiapine-XR groups (−11.02 [11.06] and −10.74 [6.97], respectively; LS mean difference [80% CI]: 0.38 [−2.46, 3.22]) (Supplementary Table 2). However, PROMIS-SRI T-score change from baseline to week 24 was more prominent for the seltorexant 20-mg MDG (−14.7 [11.32]) than the 40-mg MDG (−8.6 [10.49]) or the combined quetiapine-XR group (−10.74 [6.97]).

Similar to MADRS findings, PROMIS-SD and PROMIS-SRI T-scores consistently improved over time in the seltorexant 20-mg MDG versus the 40-mg MDG and combined quetiapine-XR group (Table 2**,**  Supplementary Table 2). Further, reduction in mean (SD) PROMIS-SD raw scores was numerically greater at week 24 in the seltorexant 20-mg MDG (−14.3 [9.71]) than the quetiapine-XR group (−11.2 [6.70]).

#### Other endpoints

Based on HAM-A total score, anxiety-related symptom severity improved over time and was comparable for patients in the seltorexant and quetiapine-XR groups (Supplementary Table 2). In addition, patient-reported symptoms associated with MDD generally declined over time in both treatment groups.

Patients in the quetiapine-XR group showed greater improvement in mean SMDDS scores than patients in the seltorexant group starting at week 12 and throughout DBT. Difference in LS means (80% CI) between the seltorexant and quetiapine-XR group at week 24 was 3.6 (−0.1, 7.2) (Supplementary Table 2). Finally, patients in both treatment groups generally had a gradual and similar, but not significantly different improvement in depression severity over time based on the PGI-S (Supplementary Table 2).

#### Quality of life and clinical global impression-severity

No significant difference between treatment groups was detected in CGI-S or QLDS. Scores generally improved gradually over time for both treatments, with mean CGI-S scores improving slightly numerically more for quetiapine-XR versus seltorexant (Supplementary Table 2).

#### Cognitive function

Improvement in SDMT total scores started at week 6 in the combined seltorexant group and was maintained throughout DBT; no improvement was seen in the combined quetiapine-XR group (Supplementary Table 2). LS mean difference (80% CI) for the seltorexant vs quetiapine-XR group in change from baseline at week 24 was 2.7 (−1.2, 6.5). However, improvement in SDMT total scores at every timepoint (weeks 6, 12, and 24) was attributable to the seltorexant 20-mg MDG, in which mean (SD) change from baseline was 10.8 (22.34) compared to 0.7 (13.64) for the 40-mg MDG and 0 (9.83) for the quetiapine-XR group at week 24. Neither treatment affected TMT-Part B or Hopkins Verbal Learning Test-Revised scores (Supplementary Table 2).

#### Safety

**Figure 5:**
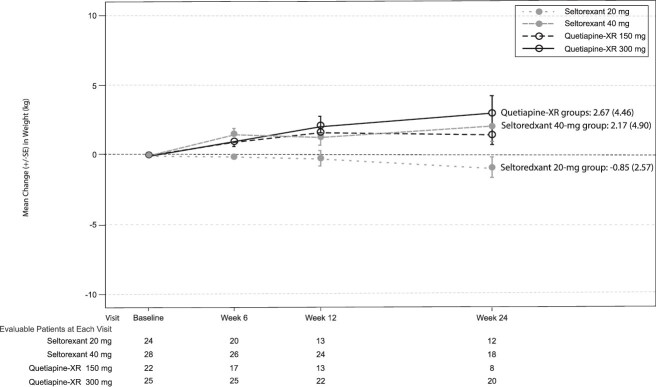
Change in weight by mode-dose group. Abbreviations: SE, standard error; XR, extended release.

Overall, 65.4% and 80.8% of patients in the seltorexant and quetiapine-XR groups, respectively, experienced ≥1 TEAE (Table 5), and most TEAEs (seltorexant, 53.8%; quetiapine-XR, 69.2%) occurred before week 6. Most common TEAEs (≥5% in either group) were abnormal dreams (13.5%), somnolence (11.5%), weight gain (7.7%), and headache, anxiety, and dry mouth (5.8% each) with seltorexant, and dry mouth (25.0%), somnolence (21.2%), fatigue (7.7%), and weight gain, bronchitis, hypersomnia, diarrhea, nausea, and vomiting (5.8% each) with quetiapine-XR. More somnolence was reported in the seltorexant 20-mg (20.8%) than 40-mg (3.6%) MDG, potentially because all patients received 20 mg for ≥14 days before increasing to 40 mg. Somnolence rates, however, were lower in the seltorexant 20-mg MDG than the quetiapine-XR 150-mg MDG (31.8%). Two patients in the seltorexant 40-mg MDG and 4 in the quetiapine-XR 300-mg MDG had ≥7% weight gain. By week 24, a mean weight increase was observed in both quetiapine-XR MDGs. Mean weight also increased for the seltorexant 40-mg MDG and was between that for the quetiapine-XR 150- and 300-mg MDGs (Figure 5). In contrast, the seltorexant 20-mg MDG showed a small, mean weight decrease.

**Table 5:** Safety summary (safety analysis set).

Characteristic	Seltorexant *n* = 52	Quetiapine-XR *n* = 52
Patients with ≥1 TEAE, *n* (%)	34 (65.4)	42 (80.8)
Most common TEAEs (≥5% of patients in either group), *n* (%)		
Abnormal dreams	7 (13.5)	1 (1.9)
Somnolence	6 (11.5)	11 (21.2)
Weight gain	3 (5.8)^a^	3 (5.8)
Headache	3 (5.8)	1 (1.9)
Anxiety	3 (5.8)	2 (3.8)
Dry mouth	3 (5.8)	13 (25.0)
Fatigue	2 (3.8)	4 (7.7)
Bronchitis	1 (1.9)	3 (5.8)
Hypersomnia	0 (0.0)	3 (5.8)
Diarrhea	0 (0.0)	3 (5.8)
Nausea	0 (0.0)	3 (5.8)
Vomiting	0 (0.0)	3 (5.8)
TEAEs leading to study drug discontinuation, *n* (%)	7 (13.5)^b^	7 (13.5)^c^
Patients with ≥1 serious TEAE, *n* (%)	1 (1.9)^d^	2 (3.8)^e^
TEAEs of special interest, *n* (%)	7 (13.5)^f^	3 (5.8)^g^

Abbreviations: TEAE, treatment-emergent adverse event; XR, extended release.

TEAEs of special interest were cataplexy, sleep paralysis, and complex sleep-related behaviors.

a One additional event in the seltorexant 20-mg mode-dose group was erroneously recorded and corrected in the table.

b Two patients reported anxiety, and 1 each reported dizziness postural and anxiety, myalgia and musculoskeletal stiffness, insomnia, abnormal dreams and confusional arousal, and tinnitus leading to study drug discontinuation.

c Two patients reported somnolence, and 1 patient each reported fatigue and somnolence; sedation; suicide attempt; bronchitis; and events of disorientation, hypersomnia, and sleep paralysis leading to study drug discontinuation.

d Chronic obstructive pulmonary disorder and atrial flutter were reported as a serious TEAE in 1 patient during the last 2 weeks of treatment.

e One patient reported worsening spinal column stenosis as a serious TEAE approximately 1 month after starting quetiapine-XR, and 1 patient was hospitalized approximately 3 months after starting quetiapine-XR following a suicide attempt by drug overdose (not study drug).

f One patient reported parasomnia and 6 reported abnormal (vivid) dreams, of whom 1 also reported confusional arousal.

g One patient each reported somnambulism, abnormal dreams, and sleep paralysis.

TEAEs led to discontinuation of 7 (13.5%) patients in each treatment group (Table 5). Three patients (seltorexant, 1 [1.9%]; quetiapine-XR, 2 [3.8%]) experienced a serious TEAE during DBT (Table 5); all were deemed not or doubtfully study drug related. One death, considered not treatment emergent nor study drug related, occurred in the seltorexant group (Figure 1). Treatment-emergent adverse events of special interest were reported by 7 (13.5%) patients in the seltorexant group and 3 (5.8%) patients in the quetiapine-XR group (Table 5).

Homeostatic model assessment to estimate changes in beta-cell function and insulin sensitivity showed geometric mean baseline Homeostatic Model Assessment for Insulin Resistance (HOMA-IR) levels of 2.00 and 1.92 (within early insulin resistance range) in the seltorexant and quetiapine-XR groups, respectively. At endpoint, values slightly decreased in the combined seltorexant group (to 1.96) but slightly increased in the combined quetiapine-XR group (by 8.8% to 2.09; Supplementary Table 3). The most remarkable treatment-associated change in metabolic laboratory parameters was in triglyceride levels, with a significant reduction from baseline to endpoint in the seltorexant group (mean [SD]: −30.5 [87.81] mg/dL; 95% CI: [−57.5, −3.5]) but not in the quetiapine-XR group (−6.4 [65.88] mg/dL; 95% CI: [−27.5, 14.7]; Supplementary Table 4).

The proportion of patients meeting criteria for sexual dysfunction—i.e., ASEX score of ≥19, or a score of ≥5 on any item, or a score of ≥4 on any 3 items—numerically decreased more from baseline to week 24 with seltorexant (baseline: overall, 80.8%; females, 91.2%; males, 61.1% vs week 24: overall, 60.0%; females, 63.2%; males, 54.5%) than quetiapine-XR (baseline: overall, 86.3%; females, 91.2%; males, 76.5% vs week 24: overall, 71.4%; females, 83.3%; males, 50.0%). Mean ASEX score also decreased more with seltorexant (baseline: overall, 22.5%; females, 24.3%; males, 19.1% vs week 24: overall, 19.2%; females, 21.2%; males, 15.7%) than quetiapine-XR (baseline: overall, 22.9%; females, 24.3%; males, 20.2%; week 24: overall, 21.0%; females, 23.5%; males, 16.6%). Further, the proportion of patients with no/very weak/somewhat weak sex drive decreased (improved) more after treatment with seltorexant (baseline: overall, 86.5%; females, 88.2%; males, 83.3% vs week 24: overall, 56.7%; females, 63.2%; males, 45.5%) than with quetiapine-XR (baseline: overall, 88.2%; females, 91.4%; males, 76.5% vs week 24: overall, 75.0%; females, 83.3%; males, 60.0%) at week 24.

Except for the metabolic laboratory measures reported above, no clinically meaningful treatment-emergent changes in chemistry, hematology, or urinalysis parameters, or vital signs and most physical findings, were observed (Supplementary Table 4). No patient reported suicidal behavior, and no clinically relevant increase in suicidal categories of suicidal ideation based on C-SSRS were observed.

## Discussion

In this study of flexibly dosed seltorexant vs flexibly dosed quetiapine-XR as adjunctive treatment to an SSRI/SNRI in patients with MDD, time to all-cause discontinuation over a 24-week period (primary endpoint) was comparable between treatments. However, discontinuations for potential treatment-related reasons were fewer for seltorexant. Depressive symptoms consistently improved over time in the seltorexant 20-mg MDG, especially in patients with higher baseline insomnia symptoms, compared with the seltorexant 40-mg MDG and combined quetiapine-XR group as evidenced by greater reductions in MADRS total score. Furthermore, no new safety signals were seen for seltorexant in this longer-term study. These and additional findings from this and other studies^14–18^ support the favorable tolerability and preliminary efficacy of seltorexant 20 mg as adjunctive treatment in patients with MDD, especially those with significant insomnia symptoms, and demonstrate potential approaches to differentiate seltorexant from quetiapine-XR.^4^^,^^5^

All-cause discontinuation rate was 47.1% with quetiapine-XR, which corroborates findings from a previous study of maintenance monotherapy to prevent relapse for MDD,^24^ and was lower (41.2%) with seltorexant. In a preplanned sensitivity analysis, fewer patients in the seltorexant (29.4%) than quetiapine-XR (43.1%) group discontinued for potentially treatment-related reasons, and time to potentially treatment-related discontinuation was longer with seltorexant (25^th^ percentile: 107 days) than quetiapine-XR (42 days). While rates of discontinuation due to TEAEs were the same for both treatments (13.5%), discontinuations for reasons such as noncompliance and lack of efficacy were higher for quetiapine-XR than seltorexant. Evaluation of all-cause discontinuation by final and MD, and for the seltorexant 20-mg–only group, showed similar trends: most discontinuations for the seltorexant 20-mg group occurred within the first 7 weeks, then leveled off over time.

Without regard to dose, numerically greater improvements in MADRS total score were observed with quetiapine-XR versus seltorexant (except at week 2); however, between-group differences were neither significantly different nor clinically meaningful.^38^ Thus, seltorexant had similar preliminary efficacy but fewer side effects than an approved adjunctive agent. In contrast, seltorexant 20-mg MDG findings suggest greater improvement and less weight gain than in the seltorexant 40-mg MDG or combined quetiapine-XR group. Change in MADRS total score with seltorexant 20 mg corroborates prior efficacy results.^14–17^^,^^39^ Additionally, the observation that seltorexant 20 mg was more effective at reducing depressive symptoms than seltorexant 40 mg over 24 weeks parallels findings from 2 short-term trials^15^^,^^16^; however, findings from these studies were not available when the current study was designed and initiated, and the flexible-dose design used herein limits comparison across doses. In this study, larger improvements in MADRS total score at week 2 (when only the lower dose was used) were observed in the seltorexant 20-mg versus the 40-mg MDGs; the smaller initial response in the 40-mg MDG may have contributed to investigators up-titrating the dose. A smaller week-2 difference was observed between quetiapine-XR 150-mg MD and 300-mg MD, and the 2 doses showed similar overall preliminary efficacy. In contrast, the seltorexant 20-mg MDG showed consistent improvement in MADRS−6 and HAM-A scores, insomnia severity ratings, and performance on the SDMT over time compared with the seltorexant 40-mg MDG and the combined quetiapine-XR group, suggesting benefit on core melancholic features (assessed by the MADRS-6), hyperarousal features (e.g., anxiety, insomnia), and cognitive impairment associated with MDD. Furthermore, more patients in the seltorexant 20-mg MDG than the 40-mg MDG or the combined quetiapine-XR group were responders and remitters at weeks 18 and 24.

Also consistent with the results of other studies,^14^^,^^15^^,^^17^^,^^39^ patients in the seltorexant 20-mg (but not 40 mg) MDG with more baseline insomnia symptoms had greater improvement in MADRS total score than those with fewer symptoms. However, subgroups in the current study were small, and results differentiated by baseline insomnia should be interpreted with caution.

Patients were randomized into the 2 treatment groups but not into individual dose groups because of the flexible-dosing design, and important covariates (eg, response to low doses) may have confounded dose choice. A discontinuation analysis by MD was confounded by patients initially receiving low-dose study drug for ≥14 days. Consequently, a patient’s dose could not reach a high MD until at least approximately day 29 of treatment. Why some patients in the seltorexant 40-mg MDG remained in the study despite limited depressive symptom improvement is unclear but conceivably may reflect the clinically meaningful improvements experienced in anxiety symptoms (HAM-A score improvements were comparable to those with quetiapine-XR). Few patients in either treatment group had a dose reduction after progressing to the high dose, likely because of comparable tolerability between high and low doses for each drug. Since short-term efficacy responses likely impacted dose selection and possibly long-term efficacy of seltorexant, a causal inference analysis using propensity score weighting of changes in MADRS total score over time was performed to adjust for factors potentially influencing dose selection (Supplemental materials). After weighting, the seltorexant 20-mg MDG still showed greater improvement at weeks 18 and 24 than the 40-mg MDG (>10-point difference) and combined quetiapine-XR group (>3-point improvement), providing further support for continued efficacy in patients who maintained 20 mg of seltorexant beyond week 6.

Overall tolerability of seltorexant was similar to that previously reported,^14–17^ with a better tolerability profile than that of quetiapine-XR based on proportion of patients with TEAEs. For seltorexant and quetiapine-XR, TEAEs generally appeared within the first 6 weeks. Somnolence rates with seltorexant were consistent with those previously observed^15^; however, the rate was higher in the 20-mg than 40-mg MDG, possibly because all patients first received 20 mg for ≥14 days before having an optional increase to 40 mg. Additionally, somnolence rates with the seltorexant 20-mg MDG were lower than for the combined quetiapine-XR and quetiapine-XR 150-mg MD groups. Discontinuations due to TEAEs and other reasons also were more common in this study than in prior seltorexant studies,^14^^,^^15^^,^^17^ potentially because of the longer treatment period.

Several TEAEs of weight gain, including 2 cases of ≥7% weight gain, were reported with the seltorexant 40-mg MD, and mean weight change was between those of the 2 quetiapine-XR MDGs. In contrast, mean weight decreased slightly (by 0.85 kg) in the seltorexant 20-mg MDG while increasing (by 2.67 kg) in the combined quetiapine-XR group. The latter result is consistent with prior reports.^7^^,^^22^^,^^23^^,^^40^ If such effects are replicated in long-term studies with larger sample sizes, the absence of weight gain and significant reduction in triglyceride levels with seltorexant 20 mg would be clinically important. MDD and obesity are related, with evidence indicating an association between diagnosed MDD and obesity incidence, and a bidirectional relationship between depressive symptoms and obesity.^41–46^ Also, weight gain with metabolic changes expected to contribute to cardiovascular disease risk has been associated with quetiapine-XR and other antipsychotics approved for adjunctive use in MDD,^7^^,^^9^^,^^23^ and low-dose quetiapine has been associated with increased risk of major adverse cardiovascular events.^8^ Results of this study further support an improved safety profile of seltorexant 20 mg compared with quetiapine-XR at its approved daily doses when used adjunctively in MDD.

This study had several strengths, including a longer treatment duration with confirmation of seltorexant safety and tolerability for longer than previously investigated. Further, stratification by baseline insomnia symptoms allowed for evaluation of preliminary seltorexant efficacy in patients with MDD and residual insomnia. This study also had some limitations. For example, based on concurrent preclinical evaluations, women of child-bearing age were excluded; however, this preclinical finding was thoroughly investigated, and this exclusion no longer applies for subsequent seltorexant clinical development. Patients with a history of intolerance or nonresponse to previous quetiapine trials were also excluded, potentially biasing the sample toward patients who previously showed tolerance or responsiveness to quetiapine (and potentially disadvantaging seltorexant by comparison). Also, the flexible-dose design may have impacted treatment course and, consequently, discontinuations, and—together with small *n*-values (22-28 patients/MDG)—limits comparison across doses.

## Conclusions

In prior seltorexant studies, the 20-mg daily dose produced a clinically meaningful reduction in depressive symptoms, particularly among those with higher baseline insomnia symptoms. This exploratory study supports the preliminary efficacy of seltorexant 20 mg daily, with fewer side effects than with quetiapine-XR, when administered adjunctively with an SSRI/SNRI to which the patient had inadequate response, especially in those with significant insomnia symptoms. Similar to the relationship between MDD and obesity, evidence suggests a bidirectional relationship and common underlying mechanisms between MDD and insomnia, which is experienced by approximately 80% of patients with MDD.^47^^,^^48^

Although all-cause discontinuation was comparable between seltorexant and quetiapine-XR (primary endpoint), discontinuations for potential treatment-related reasons were fewer with seltorexant. Depressive symptom improvement was evident at week 2 with seltorexant and increased further from weeks 6 to 24 with the seltorexant 20-mg MD, which had clinically meaningful separation from the quetiapine-XR groups on MADRS total score starting at week 18 and on response and remission rates by week 24. Overall, the tolerability profile of the seltorexant 20-mg MD was better than that of quetiapine-XR, with a potential advantage on weight change and cognitive performance. Improved cognitive performance in the seltorexant 20-mg group, in particular, may warrant further testing as a potentially specific benefit of seltorexant, since impairments in higher-order cognitive function and information processing associated with MDD reportedly persist independently of clinical symptom change in response to conventional antidepressant treatment.^49^ Finally, results demonstrate approaches to differentiate seltorexant 20 mg daily from quetiapine-XR, based on adequately powered studies with respect to response rates, remission rates, and tolerability at 6 months, reflecting a meaningful timeframe for managing the relapsing and often chronic illness course of MDD.

## Supplementary Material

Supplementary_Materials_pyag009

## Data Availability

The data-sharing policy of Johnson & Johnson is available at https://innovativemedicine.jnj.com/our-innovation/clinical-trials/transparency. As noted on this site, requests for access to the study data can be submitted through Yale Open Data Access [YODA] Project site at http://yoda.yale.edu.
