# Supplementary material for: Safety, tolerability, and preliminary efficacy of seltorexant versus quetiapine extended release as adjunctive therapy in major depressive disorder: a randomized, flexible-dose, 6-month, parallel-group, exploratory study
Source: Int J Neuropsychopharmacol. 2026 Mar 9;29(4):pyag009. doi: 10.1093/ijnp/pyag009 (PMC13070725; doi:10.1093/ijnp/pyag009)
Supplement: Supplementary_Materials_pyag009 [file supplementary_materials_pyag009.pdf]

Supplementary Materials

Safety, Tolerability, and Preliminary Efficacy of Seltorexant Versus Quetiapine Extended Release as Adjunctive Therapy in Major Depressive Disorder: A Randomized, Flexible-Dose, 6-Month, Parallel-Group, Exploratory Study

Regular research article

Christine Pinter, MS; Michael E. Thase, MD; Roger S. McIntyre, MD, FRCPC; Kimberly Cooper, MS; Haiyan Xu, PhD; Gahan Pandina, PhD; Adam Savitz, MD, PhD; and Wayne C. Drevets, MD

METHODS

Patients

Adult outpatient men or women of nonchildbearing potential aged 18-70 years (inclusive) who met Diagnostic and Statistical Manual of Mental Disorders, Fifth Edition [DSM-5] criteria for major depressive disorder (MDD) without psychotic features (confirmed by the Structured Clinical Interview for DSM-5 Axis I Disorders-Clinical Trial Version) and had inadequate response (defined as <50% improvement in depressive symptoms) to 1–3 antidepressants administered at an adequate dose and duration in the current depressive episode (based on the Massachusetts General Hospital Antidepressant Treatment Response Questionnaire), and were currently receiving treatment for depressive symptoms with 1 selective serotonin reuptake inhibitor (SSRI) or selective norepinephrine reuptake inhibitor (SNRI) antidepressant (citalopram, duloxetine, escitalopram, fluvoxamine, fluoxetine, milnacipran, levomilnacipran, paroxetine, sertraline, venlafaxine, desvenlafaxine, vilazodone, or vortioxetine), in any formulation, at a stable dose for ≥4 weeks and ≤12 months at screening were eligible for enrollment. Patients were also required to have a Montgomery-Åsberg

Depression Rating Scale (MADRS)[1] total score ≥25 at screening and no clinically significant improvement (≤20% reduction in MADRS total score) from screening to the baseline visit.

Key exclusion criteria included history or current diagnosis of a psychotic, bipolar, autism spectrum, borderline personality, or somatoform disorder, intellectual disability, or fibromyalgia; significant primary sleep disorders, including obstructive sleep apnea, restless leg syndrome, narcolepsy, or parasomnias; or previous history of intolerance or nonresponse to quetiapine as an adjunctive MDD treatment. Patients with current active DSM-5 diagnosis of obsessive-compulsive disorder, posttraumatic stress disorder, anorexia nervosa, or bulimia nervosa were excluded. Patients with current or history of serious suicidal ideation (ie, active thoughts with at least some intent to act) within the past 6 months or history of suicidal behavior within the past year (based on the Columbia Suicide Severity Rating Scale[2]) were excluded. Patients with history of moderate or severe substance or alcohol use disorder according to DSM-5 criteria within the past 6 months, or positive test result(s) for alcohol and/or drugs of abuse at screening or baseline were excluded.

Additional prohibited medications and food supplements included S-adenosyl methionine, bupropion, opiates, and mood stabilizers (eg, lithium and anticonvulsants) from ≥7 days before Day 1 until the follow-up visit; stimulants (dexamphetamine, methylphenidate, dexmethylphenidate), oral systemic steroids, appetite suppressants (eg, ephedrine), and isoxsuprine from ≥7 days before Day 1 until the follow-up visit; prescription weight-loss medications (including but not limited to orlistat, orcaserin, combination therapy with naltrexone/bupropion, liraglutide 3 mg, topiramate/phentermine [or its individual components]) or over-the-counter weight-loss therapies from Day 1 until the follow-up visit; a known moderate or strong inhibitor/inducer of cytochrome P450 (CYP)3A4 and CYP2C9 or a dual inhibitor/inducer of CYP3A4 and CYP2C9 within 14 days (or after washout) before the first study drug administration on Day 1 until the follow-up visit; and St. John's wort, ephedra, Chinese herbal medications, ginkgo, ginseng, or kava from ≥7 days before Day 1 until the follow-up visit. Limited use of decongestants was permitted, as needed, but could not exceed 7 consecutive days or be used within 24 hours of a scheduled visit.

Supplementary Figure 1 Study drug dosing.

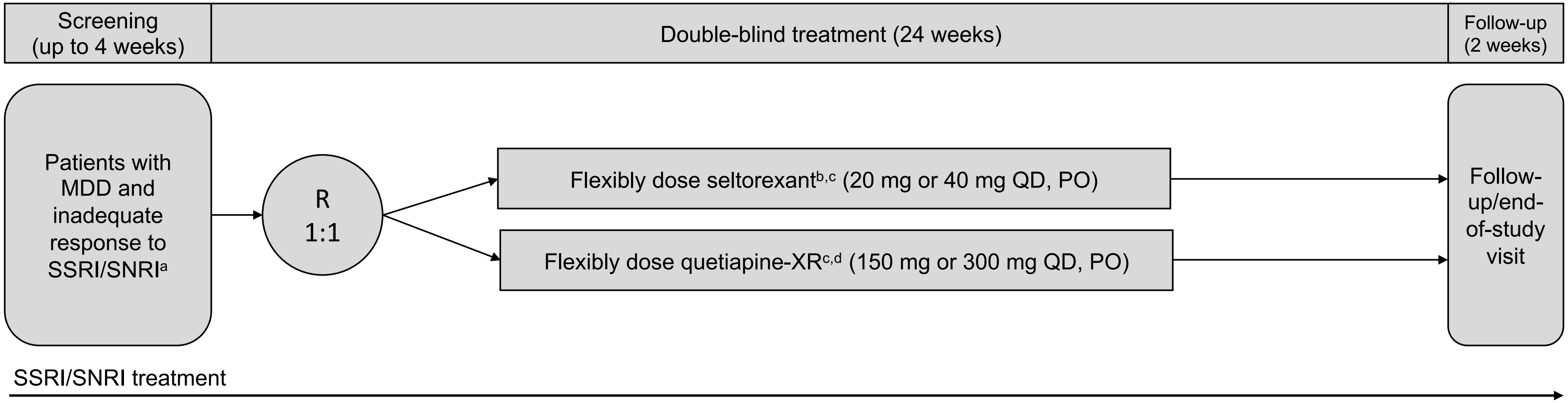

Abbreviations: MDD, major depressive disorder; PO, orally; QD, once daily; R, randomization; SNRI, serotonin-norepinephrine reuptake inhibitor; SSRI, selective serotonin reuptake inhibitor; XR extended release.

[a]In the current depressive episode, including at the time of screening. Patients continued taking their SSRI/SNRI throughout the study at the same dose and time as before study enrollment.

[b]Patients randomized to seltorexant received 20 mg as a starting dose.

[c]Patients randomized to quetiapine-XR received 50 mg for 2 days, then 150 mg on Day 3, per prescribing guidelines.

[d]After the initial dosing period, dose adjustments could have been made starting with the first scheduled clinic visit (Day 14). The first dose adjustment must have been upwards. Subsequent adjustments could have been made upwards or downwards within the study dose ranges, if necessary, depending on the investigator’s assessment of the patient’s clinical response and tolerability.

Efficacy and safety assessments

Supplementary Table 1 Secondary efficacy endpoints,[a] and safety and tolerability assessments.

	Secondary efficacy endpoint and description of assessments
MADRS	• Change from baseline over time in MADRS total score, including analyses by baseline ISI total score (≥15 vs <15) and by mode dose (daily dose taken most frequently during the study by a study patient)<br>• MADRS[1] is a scale that includes 10 core symptoms (apparent sadness, reported sadness, inner tension, reduced sleep, reduced appetite, concentration difficulties, lassitude, inability to feel, pessimistic thoughts, and suicidal thoughts), which are scored from 0 (item not present or normal) to 6 (severe or continuous presence of the symptoms). Total score ranges from 0–60, with higher scores indicates more severe depression
MADRS-6	• Change from baseline over time in the MADRS-6 score<br>o MADRS-6[3] is a subscale of MADRS that includes 6 core symptoms (apparent sadness, reported sadness, inner tension, lassitude, inability to feel, and pessimistic thoughts). Total score ranges from 0–36, and higher scores represent a more severe condition
Response	• Proportion of patients who achieved response (≥50% improvement on the MADRS from baseline) over time

	• Sustained response: Proportion of patients who achieved response at Week 12 that was sustained at Weeks 18 and 24
Remission	• Proportion of patients who achieved remission (MADRS total score ≤12) over time<br>• Sustained remission: Proportion of patients who achieved remission (MADRS total score ≤12) at Week 12 that was sustained at Weeks 18 and 24
Sleep disturbance and impairment	• Change from baseline to Weeks 12 and 24 in PROMIS-SD T-scores and raw scores and PROMIS-SRI Short Form 8a<br>o PROMIS-SD[4] is an 8-item questionnaire used to assess concepts of sleep initiation (2 items), quality of sleep (3 items), early morning feelings (2 items), and worrying about sleep (1 item). Responses are captured using a 5-point Likert scale<br>o PROMIS-SRI[4] is an 8-item questionnaire used to assess consequences of poor sleep. Responses are captured using a 5-point Likert scale. Total raw score ranges from 8-40, and higher scores denote greater levels of daytime fatigue<br>o For both PROMIS-SD and PROMIS-SRI, total raw scores range from 8–40, and higher scores denote worse sleep and sleep-related impairment, respectively. Total raw scores can be converted into T-scores, where raw scores are rescaled into standardized scores (mean [SD]: 50 [10]) for each patient

Disease severity and related symptoms	• Change from baseline to Weeks 12, 18, and 24 in the HAM-A total score<br>○ HAM-A[5] is a 14-item questionnaire used to assess the severity of different anxiety-related symptoms. However, the original HAM-A lacks instructions for administration and clear anchor points for assignment of severity ratings. Therefore, the structured interview guide version,[6] which has high inter-rater and test-retest reliability and produces similar but consistently higher scores, was used in this study.<br>○ Each of the 14 items is scored on a 5-point scale, ranging from 0 (not present) to 4 (very severe, symptom is incapacitating). Total scores range from 0–56, and higher scores denote more severe symptoms, where 0–13 indicates normal range, 14–17 indicates mild severity, 18–24 mild to moderate severity, 25–30 moderate to severe, and ≥31 severe. Higher scores represent a more severe condition<br>• Change from baseline to Weeks 12 and 24 in SMDDS, CGI-S, and PGI-S<br>○ SMDDS[7,8] is 16-item scale used to assess patient-reported symptoms associated with MDD. Each of the 16 items is scored on a 4-point scale. Before summing the items to create a total score, items 11 (how often did you have a poor appetite) and 12 (how often did you over eat) are combined by selecting the highest severity on either item. Total score,

	which ranges from 0–60, is created by summing the responses on the 15 items. Higher scores indicate more severe depressive symptomatology<br>○ CGI-S[9] is scale used to obtain a global evaluation of the patient's condition at a given time and evaluate the severity of psychopathology. The scale is 1–7, where higher scores indicate more severe illness<br>○ PGI-S[10] is a scale to measure severity of illness. The scale is 1–4, where higher scores indicate more severe illness
Quality of life	• Change from baseline to Weeks 12 and 24 in QLDS<br>○ QLDS[11] is a disease specific, 34-item questionnaire used to assess HRQoL. Each statement is given a score of 1 or 0. Score of 1 is indicative of adverse QoL. All item scores are summed to give a total score ranging from 0 (good QoL) to 34 (very poor QoL)
Cognitive function	• Change from baseline to Weeks 6, 12, and 24 in SDMT, TMT-Part B, and HVLT-R<br>○ SDMT[12] is a paper-and-pencil assessment of complex scanning and visual tracking, where the ability to pair 9 abstract symbols with specific numbers (1–9) is assessed by tallying the number of correct substitutions within 90 seconds. This test is sensitive to impairments in cognitive function associated with MDD

	○ TMT-Part B[13] is a paper-and-pencil assessment of divided attention and executive function, where the time to pair sets of 12 consecutive numbers and 12 consecutive letters in alternating alphanumeric sequencing is assessed. This test is sensitive to cognitive decline associated with MDD[14]<br>○ HVLT-R[15] is a recall test used to measure verbal learning and memory. Administration includes 3 learning trials, a delayed (20-minute) recall trial, and a 24-word recognition list (including 12 target and 12 foil words). Scores are derived for total recall (raw number of correct responses for 3 learning trials), delayed recall (raw number of correct responses), retention (raw number of true-positive errors); and recognition and discrimination index raw number of true-positives minus number of false positives), and t scores are calculated
Safety and tolerability assessments	• Emergence of suicidal ideation was assessed using the C-SSRS, and sexual functioning was assessed using the ASEX<br>○ C-SSRS[2] is a standard measure for suicidal ideation that includes 4 constructs: severity of ideation (5-point ordinal scale), intensity of ideation (5 items, each with a 5-point ordinal scale), behavior (nominal scale), and lethality (6-point ordinal scale)

- ASEX[16] is a 5-item rating scale that quantifies sex drive, arousal, vaginal lubrication/penile erection, ability to reach orgasm, and satisfaction from orgasm. Scores range from 5–30, with higher scores indicating more sexual dysfunction

---

Abbreviations: ASEX, Arizona Sexual Experience Scale; CGI, Clinical Global Impression-Severity; C-SSRS, Columbia -Suicide Severity Rating Scale; HAM-A, Hamilton Anxiety Rating Scale; HRQoL, health-related quality of life; HVLT-R, Hopkins Verbal Learning Test-Revised; MADRS, Montgomery-Åsberg Depression Rating Scale; MDD, major depressive disorder; PGI-S, Patient Global Impression-Severity; PROMIS-SD, Patient Reported Outcome Measurement Information System-Sleep Disturbance; PROMIS-SRI, Patient Reported Outcome Measurement Information System Sleep-Related Impairment Short Form; QLDS, Quality of Life in Depression Scale; SD, standard deviation; SDMT, Symbol Digit Modalities Test; SMDDS, Symptoms of Major Depressive Disorder Scale; TMT-Part B, Trail Making Test-Part B; XR, extended release.
MADRS and CGI-S were administered by independent, centralized remote raters; the HAM-A, SDMT, TMT-Part B, HVLT-R, and C-SSRS were administered by the investigators or designee at the sites; and the PGI-S, PROMIS-SD, PROMIS-SRI, SMDDS, QLDS, and ASEX were completed directly by the patients.
Change from baseline to Week X in each parameter was calculated as (score at Week X – baseline score).

Sample size determination and analysis sets

For comparison of time to all-cause discontinuation of study drug between groups, a 1-sided log-rank test with an overall sample size of 100 patients (seltorexant: n = 50; quetiapine-XR: n = 50) provided approximately 92% power at a 1-sided 0.10 significance level to detect a hazard ratio of 0.415, assuming the proportion of patients who discontinued from study drug in the quetiapine group was 50%. The all-randomized analysis set included patients who were randomized regardless of whether study drug was received. The full analysis set was planned to include all randomized patients who received ≥1 dose of study drug. The safety analysis set included all randomized patients who received ≥1 dose of study drug.

Post hoc causal inference analysis

A causal inference analysis was performed, adjusting for potential bias in estimates of mode-dose (MD) treatment effects (MADRS total score change from baseline over time) using propensity score weighting. The approach used patient weighting based on propensity scores obtained by a generalized boosted regression model using the following factors: Week-2 MADRS total score change from baseline, baseline MADRS total score, baseline insomnia status (ISI: <15, ≥15), treatment (seltorexant, quetiapine-XR), and underlying antidepressant type (SSRI, SNRI). To estimate the average treatment effects in MADRS total score change, weights then were applied towards the mixed-model-for-repeated-measures analysis with treatment, time, baseline insomnia status, and time-by-treatment interaction as factors and baseline MADRS total score as a covariate.

Results

Primary Efficacy Endpoint

Supplementary Figure 2 Time to all-cause discontinuation of study drug by baseline ISI status (full analysis set).

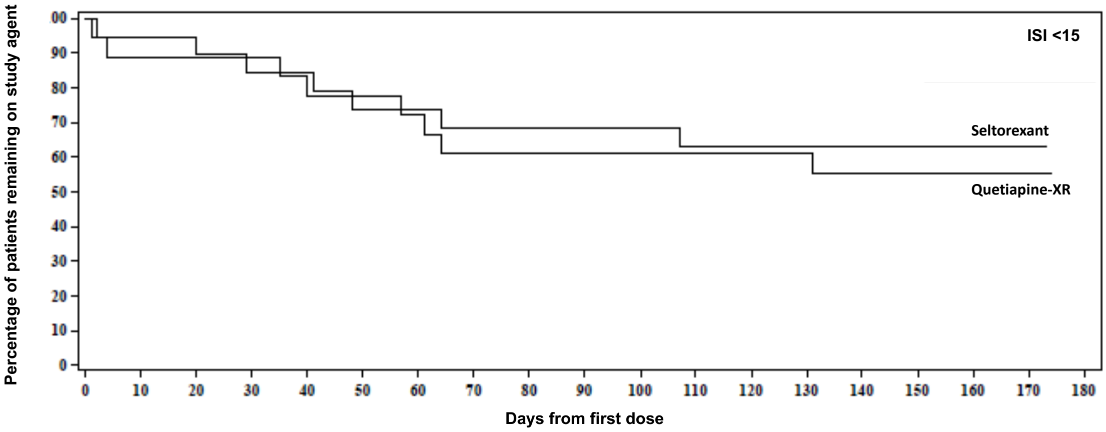

Patients at risk

Seltorexant	19	18	18	16	16	14	14	13	13	13	13	12	12	12	12	12	12	3	0
Quetiapine-XR	18	16	16	16	15	14	13	11	11	11	11	11	11	11	10	10	10	2	0

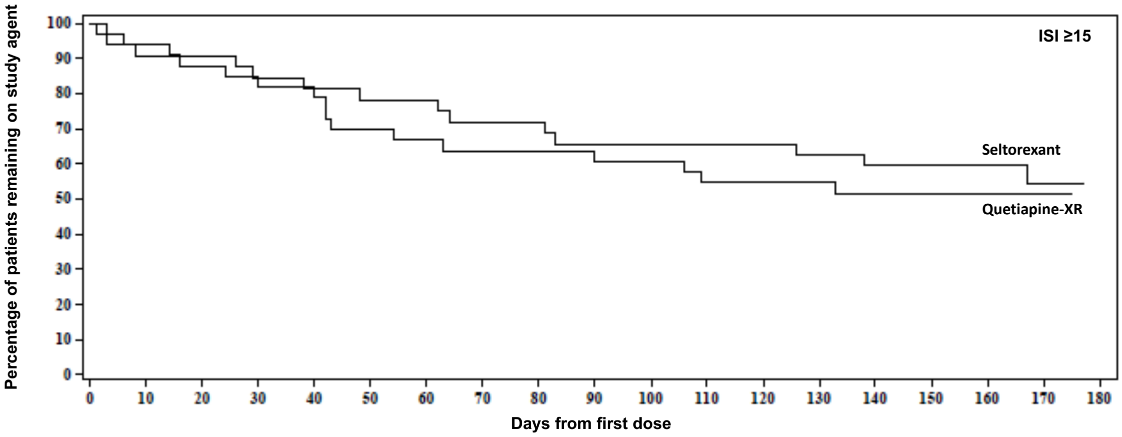

Patients at risk

Seltorexant	32	29	29	27	26	25	25	23	23	21	21	21	21	20	19	19	19	5	0
Quetiapine-XR	33	31	29	28	27	25	22	21	21	21	20	18	18	18	17	17	17	2	0

Abbreviations: ISI, Insomnia Severity Index; XR, extended release.

Secondary Efficacy Endpoints

Supplementary Table 2 Secondary efficacy endpoint results (full analysis set).

	Seltorexant				Seltorexant	Quetiapine-XR
	n	20-mg MD	n	40-mg MD	n = 51	n = 51
Sleep disturbances and impairment						
PROMIS-SRI T-score						
Baseline, mean (SD)	23	58.6 (6.99)	28	60.6 (7.36)	59.71 (7.19)	60.62 (5.89)
Change from baseline to Week 12, mean (SD)[a]	13	-13.0 (10.49)	24	-4.2 (7.35)	-7.25 (9.45)[e]	-9.09 (7.99)[e]
LS mean difference at Week 12 (80% CI)[a,b]					1.09 (-1.29, 3.46)[e]	
Change from baseline to Week 24, mean (SD)[a]	12	-14.7 (11.32)	18	-8.6 (10.49)	-11.02 (11.06)[f]	-10.74 (6.97)[f]
LS mean difference at Week 24 (80% CI)[a,b]					0.38 (-2.46, 3.22)[f]	
Disease severity and related symptoms						
HAM-A total score						
Baseline, mean (SD)	23	16.0 (4.41)	28	19.2 (18.5)	17.8 (6.03)	17.6 (6.63)
Change from baseline to Week 12, mean (SD)[a]	13	-9.1 (5.88)	24	-8.5 (6.25)	-8.7 (6.05)[g]	-6.6 (6.75)[g]
LS mean difference at Week 12 (80% CI)[a,b]					-1.1 (-2.7, 0.5)[g]	

LS mean difference at Week 12 (80% CI)[a,c]					-1.0 (-2.4, 0.5)[h]	
Change from baseline to Week 18, mean (SD)[a]	13	-9.7 (6.45)	21	-7.8 (6.84)	-8.5 (6.66)[i]	-10.0 (5.69)[i]
LS mean difference at Week 18 (80% CI)[a,b]					1.8 (0.1, 3.5)[i]	
LS mean difference at Week 18 (80% CI)[a,c]					1.1 (-0.5, 2.7)[h]	
Change from baseline to Week 24, mean (SD)[a]	12	-12.3 (6.20)	18	-9.9 (6.79)	-10.9 (6.55)[f]	-9.5 (7.26)[f]
LS mean difference at Week 24 (80% CI)[a,b]					-0.9 (-2.7, 0.8)[f]	
LS mean difference at Week 24 (80% CI)[a,c]					-0.5 (-2.2, 1.2)[h]	
SMDDS total score						
Baseline, mean (SD)	23	35.0 (5.88)	28	38.8 (8.22)	37.1 (7.43)	38.1 (8.33)
Change from baseline to Week 12, mean (SD)[a]	13	-17.8 (12.09)	24	-11.7 (9.84)	-13.9 (10.92)[e]	-15.7 (11.16)[e]
LS mean difference at Week 12 (80% CI)[a,b]					2.0 (-1.1, 5.1)[e]	
Change from baseline to Week 24, mean (SD)[a]	123	-22.1 (11.62)	18	-11.3 (12.43)	-15.6 (13.07)[f]	-19.6 (11.59)[f]
LS mean difference at Week 24 (80% CI)[a,b]					3.6 (-0.1, 7.2)[f]	
CGI-S						
Baseline, mean (SD)	23	4.7 (0.75)	28	4.5 (0.69)	4.6 (0.72)	4.8 (0.76)
Change from baseline to Week 12, mean (SD)[a]	13	-2.0 (1.47)	25	-0.08 (1.16)	-1.2 (1.39)[j]	-1.7 (1.23)[j]
Week 12, 2-sided p-value[k]					0.140	

Change from baseline to Week 24, mean (SD)[a]	12	-2.5 (1.17)	18	-0.7 (1.56)	-1.4 (1.65)[f]	-1.7 (1.10)[f]
Week 24, 2-sided p-value[k]		--		--	0.472	
PGI-S						
Baseline, mean (SD)	23	2.7 (0.62)	28	2.6 (0.74)	2.7 (0.68)	2.7 (0.66)
Change from baseline to Week 12, mean (SD)[a]	13	-1.5 (1.27)	24	-0.8 (0.85)	-1.0 (1.07)[g]	-1.1 (1.02)[g]
Week 12, 2-sided p-value[k]					0.715[g]	
Change from baseline to Week 24, mean (SD)[a]	12	-1.8 (1.11)	18	-0.8 (0.92)	-1.2 (1.10)[f]	-1.5 (1.09)[f]
Week 24, 2-sided p-value[k]					0.321[f]	
Quality of life						
QLDS total score						
Baseline, mean (SD)	23	21.7 (6.29)	28	18.6 (8.78)	20.0 (7.83)	22.1 (7.02)
Change from baseline to Week 12, mean (SD)[a]	13	-12.9 (8.42)	24	-5.5 (8.68)	-8.1 (9.20)[l]	-8.3 (8.68)[l]
LS mean difference at Week 12 (80% CI)[a,b]					-0.1 (-2.6, 2.4)[l]	
Change from baseline to Week 24, mean (SD)[a]	12	-14.9 (7.23)	18	-5.9 (10.66)	-8.7 (9.93)[f]	-9.9 (10.43)[f]
LS mean difference at Week 24 (80% CI)[a,b]					0.1 (-2.9, 3.0)[f]	
Cognitive function						
SDMT total score						

Baseline, mean (SD)	23	33.7 (18.56)	28	43.0 (15.88)	38.8 (17.59)	44.5 (15.72)
Change from baseline to Week 6, mean (SD)[d]	16	10.5 (16.30)	25	1.6 (12.23)	5.0 (14.45)[m]	-2.9 (11.72)[m]
LS mean difference at Week 6 (80% CI)[b,d]					5.8 (2.6, 8.9)[m]	
Change from baseline to Week 12, mean (SD)[b]	13	11.3 (24.04)	23	2.2 (12.12)	5.5 (17.61)[n]	0.1 (14.07)[n]
LS mean difference at Week 12 (80% CI)[b,d]					3.3 (-1.0, 7.5)[n]	
Change from baseline to Week 24, mean (SD)[b]	12	10.8 (22.34)	18	0.7 (13.64)	4.7 (18.78)[f]	0 (9.83)[f]
LS mean difference at Week 24 (80% CI)[b,d]					2.7 (-1.2, 6.5)[f]	
TMT-Part B						
Baseline, mean (SD)	23	2.0 (3.52)	28	1.0 (1.75)	1.5 (2.72)	1.2 (1.79)
Change from baseline to Week 6, mean (SD)[a]	16	-0.4 (3.98)	25	0.8 (2.69)	0.4 (3.26)[m]	0 (2.59)[m]
LS mean difference at Week 6 (80% CI)[a,b]					0.5 (-0.3, 1.3)[m]	
Change from baseline to Week 12, mean (SD)[a]	13	-1.3 (3.75)	23	1.1 (2.91)	0.2 (3.39)[n]	0.6 (2.93)[n]
LS mean difference at Week 12 (80% CI)[a,b]					-0.2 (-1.1, 0.6)[n]	
Change from baseline to Week 24, mean (SD)[a]	12	-1.2 (5.41)	18	1.6 (3.62)	0.5 (4.54)[f]	-0.3 (1.54)[f]
LS mean difference at Week 24 (80% CI)[a,b]					1.3 (0.3, 2.2)[f]	
HVLT-R						
Total recall						

Baseline, mean (SD)	23	23.4 (5.72)	28	22.4 (4.29)	22.8 (4.96)	23.0 (4.92)
Change from baseline to Week 6, mean (SD)[d]	16	1.3 (4.90)	25	0.2 (4.45)	0.6 (4.60)[m]	-0.1 (5.51)[m]
Change from baseline to Week 12, mean (SD)[d]	13	2.3 (5.78)	23	1.7 (5.02)	1.9 (5.24)[o]	1.3 (5.25)[o]
Change from baseline to Week 24, mean (SD)[d]	12	2.6 (6.75)	18	1.6 (4.07)	2.0 (5.22)[f]	1.4 (5.09)[f]
Delayed recall						
Baseline, mean (SD)	23	7.7 (2.23)	28	7.8 (2.70)	7.7 (2.47)	7.2 (2.53)
Change from baseline to Week 6, mean (SD)[d]	16	0.6 (2.34)	24	-0.1 (1.87)	0.2 (2.07)[p]	0.6 (2.57)[p]
Change from baseline to Week 12, mean (SD)[d]	13	1.7 (2.59)	23	-0.2 (2.28)	0.5 (2.54)[o]	1.0 (2.52)[o]
Change from baseline to Week 24, mean (SD)[c]	12	2.6 (2.54)	18	0.4 (2.59)	1.3 (2.74)[f]	1.4 (2.39)[f]
Total true-positive errors						
Baseline, mean (SD)	23	11.0 (1.46)	28	11.0 (3.92)	11.0 (3.04)[q]	11.5 (3.97)[q]
Change from baseline to Week 6, mean (SD)[d]	15	1.5 ( )	24	-0.1 (4.26)	0.5 (4.21)[r]	-0.4 (2.71)[r]
Change from baseline to Week 12, mean (SD)[d]	13	0.7 (1.75)	23	-0.1 (4.84)	0.2 (3.99)[o]	-1.0 (3.59)[o]
Change from baseline to Week 24, mean (SD)[d]	12	-0.3 (3.55)	17	1.1 (2.59)	0.5 (3.03)[s]	-0.7 (3.07)[s]
Recognition discrimination index						
Baseline, mean (SD)	23	10.0 (1.89)	28	10.4 (4.05)	10.2 (3.23)[q]	9.7 (5.04)[q]
Change from baseline to Week 6, mean (SD)[d]	15	2.3 (3.59)	24	-0.1 (4.08)	0.8 (4.03)[r]	1.0 (3.80)[r]

Change from baseline to Week 12, mean (SD)[d]	13	1.5 (2.26)	23	-1.2 (5.40)	-0.3 (4.67)[o]	0.1 (5.29)[o]
Change from baseline to Week 24, mean (SD)[d]	12	0.5 (3.45)	17	1.1 (2.55)	0.9 (2.91)[s]	-0.3 (4.69)[s]

Abbreviations: ANCOVA, analysis of covariance; CGI, Clinical Global Impression-Severity; CI, confidence interval; HAM-A, Hamilton Anxiety Rating Scale; HVLT-R, Hopkins Verbal Learning Test-Revised; LS, least squares; MD (mode dose), most frequently received daily dose received by a patient during the study; PGI-S, Patient Global Impression-Severity; PROMIS-SRI, Patient Reported Outcome Measurement Information System Sleep-Related Impairment Short Form; QLDS, Quality of Life in Depression Scale; SD, standard deviation; SDMT, Symbol Digit Modalities Test; SMDDS, Symptoms of Major Depressive Disorder Scale; TMT-Part B, Trail Making Test-Part B; XR, extended release.

Baseline measurement = closest measurement taken before or at the time of the first study-drug dose.

PROMIS-SRI T-score rescales the raw score into a standardized score with a mean of 50 and an SD of 10.

[a]Negative change in score indicates improvement.

[b]Based on mixed-model-for-repeated-measures model with treatment (quetiapine-XR and seltorexant treatment groups), time, baseline insomnia status, and time-by-treatment interaction as factors, and baseline score as a covariate.

[c]ANCOVA based on last observation carried forward. Negative change in score indicates improvement.

[d]Positive change in score indicates improvement.

[e]Selorexant, n = 37; quetiapine, n = 36.

[f]Selorexant, n = 30; quetiapine, n = 27.

[g]Selorexant, n = 37; quetiapine, n = 33.

[h]Selorexant, n = 42; quetiapine, n = 39.

[i]Selorexant, n = 34; quetiapine, n = 29.

[j]Selorexant, n = 38; quetiapine, n = 35.

[k]Test for no difference between treatments from ANCOVA model on ranks with treatment (seltorexant and quetiapine-XR treatment groups) and baseline insomnia status as factors, and baseline value (unranked) as a covariate (type III SS).

[l]Selorexant, n = 37; quetiapine, n = 35.

[m]Selorexant, n = 41; quetiapine, n = 39.

[n]Selorexant, n = 36; quetiapine, n = 32.

[o]Selorexant, n = 36; quetiapine, n = 31.

[p]Selorexant, n = 40; quetiapine, n = 39.

[q]Selorexant, n = 51; quetiapine, n = 50.

[r]Selorexant, n = 39; quetiapine, n = 38.

[s]Selorexant, n = 29; quetiapine, n = 26.

Safety Endpoints

Supplementary Table 3 Insulin sensitivity and beta-cell function (safety analysis set).

		Seltorexant		Quetiapine-XR
	n	n = 52	n	n = 52
HOMA-IR, mean (SD)[a]				
Baseline	50	2.66 (2.848)	50	3.26 (4.579)
Endpoint	42	3.01 (4.843)	39	3.08 (2.801)
Change, mean (SD)	40	0.51 (4.898)	37	-0.57 (4.074)
95% CI		(-1.1, 2.1)		(-1.9, 0.8)
HOMA-IR, geometric mean (SD)[a,b]				
Baseline	50	2.00 (0.98 – 4.07)	50	1.92 (0.73 – 5.07)
Endpoint	42	1.96 (0.88 – 4.38)	39	2.09 (0.83 – 5.26)
HOMA-%B, mean (SD)[a]				
Baseline	50	137.60 (138.580)	50	136.51 (140.565)
Endpoint	42	134.02 (129.906)	39	131.54 (122.645)
Change, mean (SD)	40	-8.90 (176.070)	37	-15.89 (149.461)

95% CI		(-65.2, 47.4)		(-65.7, 33.9)
HOMA-%B, geometric mean (SD)[a,b]				
Baseline	50	99.71 (45.89 – 216.64)	50	97.85 (44.30 – 216.17)
Endpoint	42	98.55 (45.26 – 214.59)	39	95.30 (42.69 – 212.73)

Abbreviations: CI, confidence interval; HOMA-IR, Homeostatic Model Assessment for Insulin Resistance; HOMA-%B, Homeostatic Model Assessment for Beta Cell Function; SD, standard deviation; XR, extended release.

[a]Only insulin and glucose levels collected in a fasting state were included in determination of HOMA-IR and HOMA-%B.

The time-point n-values are the number of patients with a non-missing value for the laboratory test at the specified time point, and the n-values for change from baseline are the number of patients with non-missing values at both baseline and postbaseline time points.

[b]Geometric mean: exp(mean(logs) - 1*SD(logs)), exp(mean(logs) + 1*SD(logs)).

Baseline = closest measurement taken before or at the time of the first study-drug dose.

End point = last measurement within the double-blind treatment phase.

Supplementary Table 4 Change from baseline over time in metabolic laboratory parameters (safety analysis set).

	Seltorexant							
		20-mg MD		40-mg MD		Seltorexant		Quetiapine-XR
	n	n = 24	n	n = 28	n	n = 52	n	n = 52
Glucose (mg/dL),[a] mean (SD)								
Baseline	24	94.3 (11.64)	28	97.5 (11.21)	52	96.0 (11.41)	52	96.5 (15.55)
Change from baseline to Week 24	11	-0.4 (11.09)	18	-4.9 (14.15)	29	-3.2 (13.06)	27	0.7 (14.07)
95% CI		(-7.8, 7.1)		(-12.0, 2.1)		(-8.2, 1.8)		(-4.9, 6.3)
Change from baseline to endpoint	17	0.1 (14.44)	26	0.5 (23.64)	43	0.4 (20.30)	40	3.5 (16.59)
95% CI		(-7.3, 7.5)		(-9.0, 10.1)		(-5.9, 6.6)		(-1.8, 8.8)
Triglycerides (mg/dL),[b] mean (SD)								
Baseline	24	144.8 (92.01)	28	144.5 (94.53)	52	144.7 (92.46)	52	141.6 (68.58)
Change from baseline to Week 24	17	-34.2 (85.89)	18	-19.0 (101.80)	29	-28.9 (95.36)	27	-11.1 (52.60)
95% CI		(-103, 12.6)		(-69.6, 31.6)		(-65.2, 7.4)		(-31.9, 9.7)
Change from baseline to endpoint	17	-34.2 (78.06)	26	-28.1 (95.07)	43	-30.5 (87.81)	40	-6.4 (65.88)
95% CI		(-74.3, 6.0)		(-66.5, 10.3)		(-57.5, -3.5)		(-27.5, 14.7)

Total cholesterol (mg/dL),[c] mean (SD)								
Baseline	24	204.5 (42.00)	28	207.2 (37.09)	52	205.9 (39.06)	52	200.7 (37.86)
Change from baseline to Week 24	11	-16.5 (27.33)	18	-4.7 (22.64)	29	-9.2 (24.74)	27	-5.9 (24.24)
95% CI		(-34.9, 1.8)		(-15.9, 6.6)		(-18.6, 0.2)		(-15.5, 3.7)
Change from baseline to endpoint	17	-12.0 (29.53)	26	-4.5 (20.42)	43	-7.4 (24.38)	40	-3.6 (22.44)
95% CI		(-27.2, 3.2)		(-12.7, 3.8)		(-14.9, 0.1)		(-10.8, 3.6)
LDL cholesterol (mmol/L), mean (SD)								
Baseline	24	3.042 (0.9590)	28	3.093 (0.8236)	52	3.069 (0.8801)	52	2.891 (0.7634)
Change from baseline to Week 24	11	-0.304 (0.5943)	18	-0.052 (0.5089)	29	-0.148 (0.5466)	27	-0.025 (0.5510)
95% CI		(-0.7, 0.1)		(-0.3, 0.2)		(-0.4, 0.1)		(-0.2, 0.2)
Change from baseline to endpoint	17	-0.221 (0.5886)	26	-0.042 (0.4521)	43	-0.113 (0.5114)	40	-0.026 (0.4296)
95% CI		(-0.5, 0.1)		(-0.2, 0.1)		(-0.3, 0.0)		(-0.2, 0.1)

Abbreviations: CI, confidence interval; LDL, low density lipoprotein; MD, mode dose, most frequently received daily dose received by a patient during the study; SD, standard deviation; XR, extended release.

[a]Glucose conversion factor: 1 mg/dL = 0.05551 mmol/L.

[b]Triglycerides conversion factor: 1 mg/dL = 0.01129 mmol/L.

[c]Cholesterol conversion factor: 1 mg/dL = 0.02586 mmol/L.

Baseline measurement is the closest measurement taken before or at the time of the first study-drug dose.

The n-value for each measured value is the number of patients with a non-missing value for the laboratory test at the specified time point. The n-value for change from baseline is the number of patients with nonmissing values at both baseline and postbaseline time points. End point values are from the last measurement within the double-blind period.

References

1. Montgomery SA, Asberg M. A new depression scale designed to be sensitive to change. *Br J Psychiatry*. Apr 1979;134:382-9. doi:10.1192/bjp.134.4.382
2. Posner K, Brown GK, Stanley B, et al. The Columbia-Suicide Severity Rating Scale: initial validity and internal consistency findings from three multisite studies with adolescents and adults. *Am J Psychiatry*. Dec 2011;168(12):1266-77. doi:10.1176/appi.ajp.2011.10111704
3. Bech P, Tanghoj P, Andersen HF, Overo K. Citalopram dose-response revisited using an alternative psychometric approach to evaluate clinical effects of four fixed citalopram doses compared to placebo in patients with major depression. *Psychopharmacology (Berl)*. Aug 2002;163(1):20-5. doi:10.1007/s00213-002-1147-6
4. Yu L, Buysse DJ, Germain A, et al. Development of short forms from the PROMIS sleep disturbance and Sleep-Related Impairment item banks. *Behav Sleep Med*. Dec 28 2011;10(1):6-24. doi:10.1080/15402002.2012.636266
5. Hamilton M. The assessment of anxiety states by rating. *Br J Med Psychol*. 1959;32(1):50-5. doi:10.1111/j.2044-8341.1959.tb00467.x
6. Shear MK, Vander Bilt J, Rucci P, et al. Reliability and validity of a structured interview guide for the Hamilton Anxiety Rating Scale (SIGH-A). *Depress Anxiety*. 2001;13(4):166-78.
7. McCarrier KP, Deal LS, Abraham L, et al. Patient-centered research to support the development of the symptoms of Major Depressive Disorder Scale (SMDDS): Initial qualitative research. *Patient*. Apr 2016;9(2):117-34. doi:10.1007/s40271-015-0132-1
8. Bushnell DM, McCarrier KP, Bush EN, et al. Symptoms of Major Depressive Disorder Scale: Performance of a novel patient-reported symptom measure. *Value Health*. Aug 2019;22(8):906-915. doi:10.1016/j.jval.2019.02.010
9. Guy W. Clinical Global Impressions. *ECDEU Assessment Manual for Psychopharmacology*. U.S. Department of Health, Education, and Welfare, Public Health Service, Alcohol, Drug Abuse, and Mental Health Administration, National

Institute of Mental Health, Psychopharmacology Research Branch, Division of Extramural Research Programs; 1976:217-222:chap 028.

10. Eremenco S, Chen WH, Blum SI, et al. Comparing patient global impression of severity and patient global impression of change to evaluate test-retest reliability of depression, non-small cell lung cancer, and asthma measures. *Qual Life Res*. Dec 2022;31(12):3501-3512. doi:10.1007/s11136-022-03180-5
11. Hunt SM, McKenna SP. The QLDS: a scale for the measurement of quality of life in depression. *Health Policy*. Oct 1992;22(3):307-19. doi:10.1016/0168-8510(92)90004-u
12. Smith A. *Symbol digit modalities test*. 1973.
13. Lezak MD, Howieson DB, Loring DW. Trail Making Test (TMT). *Neuropsychological Assessment, Edition 5*. Oxford University Press; 2004:chap Orientation and Attention.
14. Snyder HR. Major depressive disorder is associated with broad impairments on neuropsychological measures of executive function: a meta-analysis and review. *Psychol Bull*. Jan 2013;139(1):81-132. doi:10.1037/a0028727
15. Benedict RHB, Schretlen D, Groninger L, Brandt J. Hopkins Verbal Learning Test-Revised: Normative data and analysis of inter-form and test-retest reliability. *Clin Neuropsychologist*. 1998;12:43-55. doi:10.1076/clin.12.1.43.1726
16. McGahuey CA, Gelenberg AJ, Laukes CA, et al. The Arizona Sexual Experience Scale (ASEX): Reliability and validity. *J Sex Marital Ther*. Jan-Mar 2000;26(1):25-40. doi:10.1080/009262300278623
